# Vibrational Analysis of Composite Conical-Cylindrical Shells with Functionally Graded Coatings in Thermal Environments

**DOI:** 10.3390/ma17184576

**Published:** 2024-09-18

**Authors:** Jinan Li, Yao Yang, Junxue Hou, Xiangping Wang, Haiyang Zhang, Haizhou Wang, Hui Li

**Affiliations:** 1 School of Mechanical Engineering and Automation, Northeastern University, Shenyang 110819, China; jinanli2022@163.com (J.L.); yaoang0716@163.com (Y.Y.); 13104221231@163.com (J.H.); 2 Key Laboratory of Impact Dynamics on Aero Engine, Shenyang 110015, China; wangpuma@163.com (X.W.); zhyu4555@163.com (H.Z.); sgw77915@163.com (H.W.); 3 Key Laboratory of Vibration and Control of Aero-Propulsion Systems Ministry of Education of China, Northeastern University, Shenyang 110819, China

**Keywords:** functionally graded coatings, thermal environments, conical-cylindrical shells, thermal vibration test, vibration suppression

## Abstract

This article studies the vibrational behavior of composite conical-cylindrical shells (CCSs) with functionally graded coatings (FGCs) in thermal environments using the first-order shear deformation theory. Firstly, the equivalent material parameters, fundamental frequency, and resonant displacement responses of the CCSs with FGCs are derived using the mixture principle, complex modulus method, and transfer function approach. Then, detailed thermal vibration tests are performed on CCS structures with and without coatings to assess the reliability of the proposed model, revealing that the current model accurately forecasts the thermal vibration behavior of the CCSs with FGCs. Finally, the effect of key parameters on the vibrational properties of the CCSs with FGCs is investigated. The results demonstrate that increasing the functionally graded index, coating thickness, and Young’s modulus ratio can greatly enhance the vibration suppression capability of the structure.

## 1. Introduction

Composite conical-cylindrical shells (CCSs) are widely utilized as skins or supporting components in critical fields, including the military and aerospace industries [1,2,3], due to their superior mechanical qualities and high strength-to-weight ratio [4,5,6]. However, CCSs frequently operate in environments with several coupled dynamic loads such as temperature field, mechanical stress, and inertial load [7,8,9,10]. These environments can easily induce resonance, causing fatigue damage or even failure [11,12,13]. Therefore, it is vital to explore the vibration suppression methods of CCSs working in thermal environments.

Currently, vibration suppression methods for composite plate and shell structures have garnered much attention, and many researchers are committed to exploring more effective vibration reduction strategies. In recent years, researchers have adopted the method of embedding viscoelastic cores inside structures or attaching viscoelastic materials to the surface to reduce vibration [14,15,16,17,18,19,20,21,22,23,24]. These methods utilize the properties of viscoelastic materials and can efficiently absorb and disperse vibration energy. However, the properties of viscoelastic materials are highly sensitive to temperature and vibration frequency, which may lead to a significant decrease in vibration-damping effectiveness. Simultaneously, the active control of vibration by embedding piezoelectric materials inside the structure or using macroscopic fiber composite materials has also been widely discussed [25,26,27,28]. This method is favored for its fast response speed, precise electrical signal control ability, small size, and lightweight. However, piezoelectric materials have a restricted deformation range, making them unsuitable for high-intensity or large-scale vibration issues. In addition, some researchers have used functionally graded materials, such as carbon nanotubes [29,30,31,32], to suppress vibrations in the structural matrix. This method requires improving the structural matrix, which is not feasible for many already manufactured parts and is also difficult to achieve on parts with complex geometries. Furthermore, temperature has a significant impact on their performance, and precise models and intricate control algorithms are needed to guarantee their efficacy. To solve the above problems, this article proposes functionally graded coating (FGC) technology. FGC refers to the mixing of two different materials with different volume ratios and layering characteristics [33]. In recent years, studies have found that FGC has vibration-reduction capabilities and has become a vibration-reduction technology with great application prospects. However, during the application process, the thermal environment has a significant impact on structural performance. Therefore, it is urgent to explore a coating with functionally graded features. Ceramic coating and its mixes can effectively solve this problem. They not only maintain damping capacity in high-temperature conditions but also prevent corrosion and reduce friction [34]. To apply this vibration reduction technology, it is necessary to dynamically model the conical-cylindrical shells with FGC and analyze their vibration characteristics to achieve the purpose of predicting their vibration behavior. Therefore, this work investigates the vibrational properties of the CCSs with FGCs in thermal environments.

Researchers have studied coatings on plates, shells, and blades in great detail in the last few years. For instance, Zhang et al. [35,36] used the Rayleigh–Ritz method to investigate the nonlinear vibration properties of hard-coating cylindrical shells with base excitation and non-classical elastic limitations. Furthermore, they proposed a parameterized multi-partition method to model the arbitrary multi-ring hard coating treatment of thin-walled cylindrical shells, taking into account different numbers of rings and coating ratios and exploring how variations in ring count and coating ratio influence the vibration and damping properties of these shells. Using the reduced order finite element model, Chen et al. [37] investigated the influence of damping hard coating on the vibration response of tuned and detuned blade disks. Zhang et al. [38] created an analysis model for a rotating conical cantilever cylindrical panel with a graphene coating and explored the free vibration properties of a rotating pre-twisted tapered blade. Du et al. [39] used the domain decomposition approach and the strain energy density theory to investigate the nonlinear vibration behavior of a rotating hard-coated cylindrical shell under radial harmonic excitation. They investigated how rotation speed and excitation amplitude affect the nonlinear vibration characteristics. Furthermore, this article emphasizes the analysis of composite shell structures with FGC in thermal environments and reviews significant literature on the use of coatings for vibration analysis under such conditions.

Based on the von-Karman nonlinear theory and first-order shear deformation theory, Liew et al. [40] investigated the linear and nonlinear vibration characteristics of a three-layer cylindrical panel composed of a coating, functionally graded material (FGM), and substrate. This study considered general boundary conditions and the effects of a thickness-direction temperature gradient resulting from steady-state heat conduction. Cao et al. [41] employed classical von Karman plate theory alongside first-order shear deformation theory to develop a rotating cantilever sandwich plate mode with pre-twisted and preset angles to study the vibration behavior of wind turbine blades with thermal barrier coating (TBC) layers. Reghu et al. [42] employed atmospheric plasma spraying to apply a consistent and well-adherent thermal barrier coating to the contour of an automotive piston and performed thermal fatigue tests on the coated pistons to evaluate the properties of the coating after vibration. Vishnu et al. [43] used thermal barrier coatings on turbine blades to reduce the impacts of high temperatures, stress, and vibration on turbine blades. Li et al. [44] utilized the first-order shear deformation theory in conjunction with the Rayleigh–Ritz method to analyze the thermal vibration aging characteristics of thin-walled cylindrical shell structures equipped with functionally graded protective coatings. The findings indicate that functionally graded coatings have a positive influence on vibration aging. Sun et al. [45] employed the first-order shear deformation theory, random vibration theory, and the complex modulus method to propose a vibration fatigue life prediction method for composite plates with functionally graded coatings subjected to basic random excitation. Their research evaluated the effect of critical coating parameters on the vibration fatigue life of the composite structures, providing insights into how these factors influence performance under dynamic loading conditions.

From the literature review presented, it is evident that there is a gap in the research concerning the dynamic analysis of CCSs with FGCs in thermal environments. In addition, the analysis of key parameters of the composite shells with coating is also very rare. Therefore, it is very meaningful to conduct a vibration investigation of the structure through theoretical modeling and experimental testing and provide engineering suggestions. This paper mainly investigated the free and forced vibration of CCSs with FGCs in thermal environments. First, the theoretical model of combined shells with coatings in thermal environment is established in Section 2, and the dynamic equations for solving the vibration characteristic parameters are derived. Then, in Section 3, detailed experimental tests on specimens with and without functionally graded coating in thermal environments are carried out and the currently proposed model is reliably verified. Finally, parametric analysis is performed in Section 4, the effects of some key parameters on the structural vibration characteristics are discussed, and some significant conclusions are summarized.

The innovations of this article are described as follows: (1) the vibration suppression of composite conical-cylindrical shells with functionally graded coating in thermal environments is investigated; (2) an effective analytical model is established based on the thermal vibration experiment of the composite conical-cylindrical shells with functionally graded coating; (3) the multi-directional lamination problem of fiber-reinforced composites is considered in the theoretical model; (4) some key design recommendations for the structure are provided by considering the vibration reduction performance of the functionally graded coating.

## 2. Theoretical Formulation

In this section, the mathematical model of CCSs with FGCs is developed in a thermal environment and derives the energy equation of the structure to solve the fundamental frequency and vibration displacement response of the structure.

### 2.1. Establishment of the Model

**Figure 1:**
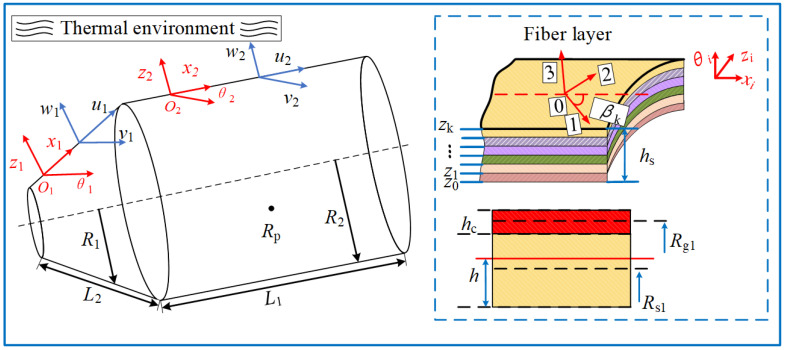
Theoretical model of conical-cylindrical shells with functionally graded coating in a thermal environment.

**Figure 2:**
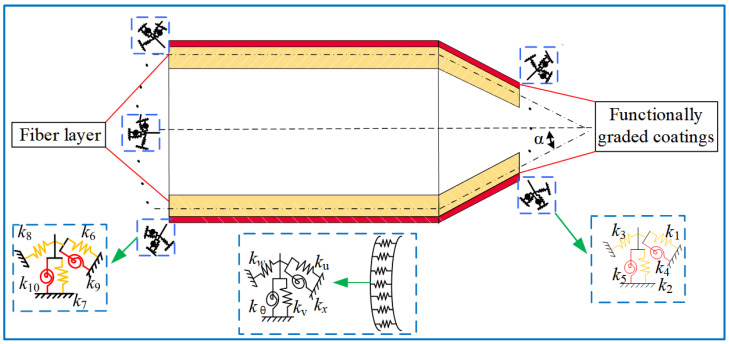
Schematic diagram of boundary and connection conditions of the coated conical-cylindrical shells.

A mathematical model of conical-cylindrical shells with functionally graded coatings in thermal environments is established, as described in Figure 1. The conical-cylindrical shells are made of fiber-reinforced material and polylactic acid resin (PLA), and the functionally graded coating evenly adheres to the surface of the conical-cylindrical shell substrate. First, two coordinate systems ($O_{1}-x_{1}\theta_{1}z_{1}$ and $O_{2}-x_{2}\theta_{2}z_{2}$) are defined on the central surface of the cylindrical and conical shells, respectively. The displacement variables ($u_{1}$, $v_{1}$, $w_{1}$) and ($u_{2}$, $v_{2}$, $w_{2}$) at any point on the composite shell structures are along the directions x, θ, and z, respectively. $R_{s1}$ and $R_{s2}$ are the small circle radii of the substrate of the cylindrical shell and the conical shell, $R_{g1}$ and $R_{g2}$ represent the attachment radii of the functionally graded layer on the cylindrical shell and the conical shell, respectively. $L_{1}$, $L_{2}$, $R_{1}$, and $R_{2}$ represent the lengths of the cylindrical and conical shells along the generatrix direction, and the equivalent radii of the small circles of the two shells, with the equivalent radius of the conical shell at any point, can be expressed as follows: $R_{a}=R_{2}+x_{1}\sin\alpha$, α is the semi-vertex angle of the conical shell. $h_{s}$ and $h_{c}$ represent the shell substrate and the functionally graded layer thickness. Also, assuming that conical-cylindrical shells consist of *N*-layer fibers of polymer. The local coordinate system established on the fiber-reinforced composite skin is 0–123, with “1”, “2”, and “3” representing directions parallel to the fiber, perpendicular to the fiber, and perpendicular to the fiber plane, respectively. Moreover, *Z*_k_ and *Z*_k−1_ represent the thickness coordinates of each fiber layer, and $\beta_{k}$ is the angle formed between the direction of fiber “1” in the *k*-th layer and the *x*-axis. In addition, the schematic diagram of boundary and connection conditions of the coated conical-cylindrical shells is shown in Figure 2, where *k*_1_, *k*_2_, *k*_3_, *k*_4_, *k*_5_, *k*_6_, *k*_7_, *k*_8_, *k*_9_, and *k*_10_ represent the boundary springs at the cylindrical end and the conical end, respectively. *k_u_*, *k_v_*, *k_w_*, *k_x_*, and *k_θ_* represent the spring stiffness of the connection between the cylindrical shell and the conical shell. It should be noted that the conical and cylindrical shells are regarded as perfectly connected, meaning that their geometric continuity and consistency in physical attributes have been maintained for the purpose of computation simplicity. Furthermore, the material parameters for each component of the CCSs with FGCs component are presented in Table 1.

Before developing the theoretical model of the shell with coating in a thermal environment, the following assumptions are made: (1) there are no adverse reactions between fiber-reinforced materials and polymer matrix; (2) the FGC and the skins of the integrated shell are securely bonded; (3) the nonlinear vibrational characteristics of CCSs and the FGC are neglected; (4) the temperature varies rapidly, neglecting the effect of short-term temperature variations on the material [46,47]; (5) the thermal moment caused by the misalignment of the neutral plane and the mid-plane due to the temperature rise is ignored.

### 2.2. Equivalent Material Parameters of Composite Material Matrix

The combined shell consists of a cylindrical shell and a truncated conical shell and is made of a mixture of matrix material polylactic acid resin (PLA) and carbon fiber, assuming that the two materials are evenly distributed. Based on the mixture principle [48], the equivalent of Young’s moduli $E_{1}^{m}$, $E_{2}^{m}$, shear modulus $G_{12}^{m},\;G_{23}^{m}$, $G_{13}^{m}$, Poisson’s ratio $\mu_{12}$, density $\rho_{1}$, and loss factors $\eta_{1}^{m}$, $\eta_{2}^{m}$, $\eta_{ij}^{m}\left(ij=12,13,23\right)$ of the CCSs can be written as follows:

(1) $$E_{1}^{m}=\left(V_{f}E_{1}^{f}+V_{p}E_{p}\right),\;\;E_{2}^{m}={\left(\frac{V_{f}}{E_{2}^{f}}+\frac{V_{p}}{E_{p}}-V_{f}V_{p}\frac{\mu_{f}^{2}E_{p}/E_{2}^{f}+\mu_{p}^{2}E_{2}^{f}/E_{p}-2\mu_{f}\mu_{m}}{V_{f}E_{2}^{f}+V_{p}E_{p}}\right)}^{-1}$$

(2) $$G_{12}^{m}=G_{23}^{m}=G_{13}^{m}={\left(\frac{V_{f}}{G_{12}^{f}}+\frac{V_{p}}{G_{p}}\right)}^{-1},\;\;\mu_{12}=V_{f}\mu_{f}+V_{p}\mu_{p},\;\;\rho_{1}=V_{f}\rho_{f}+V_{p}\rho_{p}$$

(3) $$\eta_{1}^{m}=V_{f}\eta_{1}^{f}+V_{p}\eta_{1}^{p},\;\;\eta_{2}^{m}=V_{f}\eta_{2}^{f}+V_{p}\eta_{2}^{p},\;\;\eta_{ij}^{m}=V_{f}\eta_{ij}^{f}+V_{p}\eta_{ij}^{p}\left(ij=12,13,23\right)$$

where $V_{f}$ and $V_{p}$ represent the volume fractions of the carbon fiber and PLA, respectively. Their relationship is as follows:

(4) $$\left(V_{f}+V_{p}\right)=1$$

Furthermore, the temperature-related effective material parameters of the structure are as follows:

(5) $$E_{1}^{T}=E_{1}^{m}+\left(\sum_{i=-1}^{n}a_{i}\Delta T^{i}\right),\;\;E_{2}^{T}=E_{2}^{m}+\left(\sum_{i=-1}^{n}b_{i}\Delta T^{i}\right)$$

(6) $$G_{12}^{T}=G_{12}^{m}+\left(\sum_{i=-1}^{n}c_{i}\Delta T^{i}\right),\;\;G_{13}^{T}=G_{13}^{m}+\left(\sum_{i=-1}^{n}d_{i}\Delta T^{i}\right),\;\;G_{23}^{T}=G_{23}^{m}+\left(\sum_{i=-1}^{n}e_{i}\Delta T^{i}\right)$$

(7) $$\eta_{1}^{T}=\eta_{1}^{m}+\left(\sum_{i=-1}^{n}f_{i}\Delta T^{i}\right),\;\;\eta_{2}^{T}=\eta_{2}^{m}+\left(\sum_{i=-1}^{n}g_{i}\Delta T^{i}\right),\;\;\eta_{12}^{T}=\eta_{12}^{m}+\left(\sum_{i=-1}^{n}h_{i}\Delta T^{i}\right)$$

(8) $$\eta_{13}^{T}=\eta_{13}^{m}+\left(\sum_{i=-1}^{n}j_{i}\Delta T^{i}\right),\;\;\eta_{23}^{T}=\eta_{23}^{m}+\left(\sum_{i=-1}^{n}k_{i}\Delta T^{i}\right)$$

where $E_{1}^{T}$ and $E_{2}^{T}$ represent the temperature-dependent-equivalent Young’s moduli, $G_{12}^{T},\;G_{13}^{T}$, and $G_{23}^{T}$ denote the equivalent Young’s moduli, $\eta_{1}^{T}$, $\eta_{2}^{T}$, $\eta_{12}^{T}$, $\eta_{13}^{T}$, and $\eta_{23}^{T}$ are the loss factors. $\Delta T=T-T_{0}$ is the temperature variation with regard to $T_{0}$. $a_{i},b_{i},c_{i},d_{i},e_{i},f_{i},g_{i},h_{i},j_{i}$, and $k_{i}\left(i=-1,0,1,\ldots ,n\right)$ represent a series of undetermined fitting coefficients related to the temperature.

In addition, using the complex modulus method, the equivalent complex Young’s moduli, $E_{1}^{*}$, $E_{2}^{*}$, and complex shear modulus, $G_{12}^{*},G_{13}^{*}$, and $G_{23}^{*}$, of the CCSs can be expressed as follows:

(9) $$E_{1}^{*}=E_{1}^{T}\left(1+i\eta_{1}^{T}\right),\;\;E_{2}^{*}=E_{2}^{T}\left(1+i\eta_{2}^{T}\right),\;\;G_{ij}^{*}=G_{ij}^{T}\left(1+i\eta_{ij}^{T}\right)\left(ij=12,13,23\right)$$

The axis stiffness coefficient matrix ${\bar{Q}}_{ij}^{\left(s\right)}\left(i,j=1,2,4,5,6\right)$ of the kth layer of the CCSs structure needs to be converted with the corresponding stiffness coefficient matrix $Q_{ij}^{k}\left(i,j=1,2,4,5,6\right)$. The conversion process is as follows:

(10) $${\bar{Q}}_{ij}^{\left(s\right)}=HQ_{ij}^{k}H^{T}=H\left[\begin{array}{ccccc} Q_{11}^{k} & Q_{12}^{k} & 0 & 0 & 0\\ Q_{21}^{k} & Q_{22}^{k} & 0 & 0 & 0\\ 0 & 0 & Q_{44}^{k} & 0 & 0\\ 0 & 0 & 0 & Q_{55}^{k} & 0\\ 0 & 0 & 0 & 0 & Q_{66}^{k} \end{array}\right]H^{T}$$

where H represents the transformation matrix between the local coordinate system of the *k*-th fiber layer of the CCSs and the global coordinate system, $Q_{ij}^{k}$ denotes the elements of the stiffness coefficient matrix. Their expressions can be expressed as follows:

(11) $$H=\left[\begin{array}{ccccc} \cos^{2}\beta_{k} & \sin^{2}\beta_{k} & 0 & 0 & -2\sin\beta_{k}\cos\beta_{k}\\ \sin^{2}\beta_{k} & \cos^{2}\beta_{k} & 0 & 0 & 2\sin\beta_{k}\cos\beta_{k}\\ 0 & 0 & \cos\beta_{k} & \sin\beta_{k} & 0\\ 0 & 0 & -\sin\beta_{k} & \cos\beta_{k} & 0\\ \sin\beta_{k}\cos\beta_{k} & -\sin\beta_{k}\cos\beta_{k} & 0 & 0 & \cos^{2}\beta_{k}-\sin^{2}\beta_{k} \end{array}\right]$$

(12) $$Q_{11}^{k}=\frac{E_{1}^{*}}{1-\mu_{12}\mu_{21}},\;\;Q_{12}^{k}=\frac{\mu_{12}E_{2}^{*}}{1-\mu_{12}\mu_{21}},\;\;Q_{22}^{k}=\frac{E_{2}^{*}}{1-\mu_{12}\mu_{21}},\;\;Q_{44}^{k}=G_{23}^{*}$$

(13) $$Q_{66}^{k}=G_{12}^{*},\;\;\mu_{21}=\mu_{12}\frac{E_{2}^{*}}{E_{1}^{*}},\;\;G_{12}^{*}=G_{13}^{*}=G_{23}^{*}=\frac{E_{1}^{*}}{2+2\mu_{12}}$$

### 2.3. Equivalent Material Parameters of Functionally Graded Coating

The functionally graded coating is made by combining components A and B in different proportions, where A is a ceramic coating and B is an organic solvent. According to the Voigt mixing rule [49], the effective material properties of the FGC, including Young’s modulus $E_{c}$, shear modulus $G_{c}$, Poisson’s ratio $\mu_{c}$, and density $\rho_{c}$, can be represented as follows:

(14) $$E_{c}=E_{a}V_{a}+E_{b}V_{b},\;\;G_{c}=G_{a}V_{a}+G_{b}V_{b},\;\;\mu_{c}=\mu_{a}V_{a}+\mu_{b}V_{b},\;\;\rho_{c}=\rho_{a}V_{a}+\rho_{b}V_{b}$$

where $V_{a}$ and $V_{b}$ are the volume fractions of components A and B, respectively, and their relationship can be stated as follows:

(15) $$V_{a}+V_{b}=1$$

Assuming that the material characteristics of the FGC vary with thickness and follow the law of distribution rule [50], the volume fraction $V_{b}$ can be expressed as follows:

(16) $$V_{b}={\left(\frac{z-z_{3}}{z_{2}-z_{3}}\right)}^{d}$$

where d denotes the volume gradient index of the protective coating. Figure 2 in Ref. [50] shows a detailed relationship between Young’s moduli and the functionally graded index d.

Furthermore, Young’s moduli $E_{a}^{T}$ and $E_{b}^{T}$, and loss factors $\eta_{a}^{T}$ and $\eta_{b}^{T}$ of components A and B of the FGC in the thermal environment are assumed as follows:

(17) $$E_{a}^{T}=E_{a}+\left(\sum_{i=-1}^{n}g_{i}\Delta T^{i}\right),\;\;E_{b}^{T}=E_{b}+\left(\sum_{i=-1}^{n}k_{i}\Delta T^{i}\right),\;\;\eta_{a}^{T}=\eta_{a}+\left(\sum_{i=-1}^{n}h_{i}\Delta T^{i}\right),\;\;\eta_{b}^{T}=\eta_{b}+\left(\sum_{i=-1}^{n}p_{i}\Delta T^{i}\right)$$

where $g_{i},\;k_{i},\;h_{i}$, and $p_{i}$ represent a series of temperature-dependent fitting coefficients to be determined.

According to the complex modulus approach, the equivalent complex Young’s moduli $E_{a}^{*}$ and $E_{b}^{*}$, and shear moduli $G_{a}^{*}$ and $G_{b}^{*}$ of components A and B can be stated as follows:

(18) $$E_{a}^{*}=E_{a}^{T}\left(1+j\eta_{a}^{T}\right),\;\;E_{b}^{*}=E_{b}^{T}\left(1+j\eta_{b}^{T}\right),\;\;G_{a}^{*}=\frac{E_{a}^{*}}{2\left(1+\mu_{a}\right)},\;\;G_{b}^{*}=\frac{E_{b}^{*}}{2\left(1+\mu_{b}\right)}$$

The Young’s modulus, $E_{c}^{*}$, thermal expansion coefficient $\alpha_{c}$, Poisson’s ratio $\mu_{c}$, and density $\rho_{c}$ of the mixed components A and B can be written as follows:

(19) $$E_{c}^{*}=\left(E_{b}^{*}-E_{a}^{*}\right){\left(\frac{z-z_{3}}{z_{2}-z_{3}}\right)}^{d}+E_{a}^{*},\;\;\alpha_{c}=\left(\alpha_{b}-\alpha_{a}\right){\left(\frac{z-z_{3}}{z_{2}-z_{3}}\right)}^{d}+\alpha_{a}$$

(20) $$\mu_{c}=\left(\mu_{b}-\mu_{a}\right){\left(\frac{z-z_{3}}{z_{2}-z_{3}}\right)}^{d}+\mu_{a},\;\;\rho_{c}=\left(\rho_{b}-\rho_{a}\right){\left(\frac{z-z_{3}}{z_{2}-z_{3}}\right)}^{d}+\rho_{a}$$

The axis stiffness coefficient matrix ${\bar{Q}}_{ij}^{c}\left(i,j=1,2,6\right)$ of the FGC is expressed as follows:

(21) $${\bar{Q}}_{ij}^{\left(c\right)}=\left[\begin{array}{ccc} {\bar{Q}}_{11}^{\left(c\right)} & {\bar{Q}}_{12}^{\left(c\right)} & 0\\ {\bar{Q}}_{12}^{\left(c\right)} & {\bar{Q}}_{22}^{\left(c\right)} & 0\\ 0 & 0 & {\bar{Q}}_{66}^{\left(c\right)} \end{array}\right]=\left[\begin{array}{ccc} \frac{E_{c}^{*}}{1-{\mu_{c}}^{2}} & \frac{E_{c}^{*}\mu_{c}}{1-{\mu_{c}}^{2}} & 0\\ \frac{E_{c}^{*}\mu_{c}}{1-{\mu_{c}}^{2}} & \frac{E_{c}^{*}}{1-{\mu_{c}}^{2}} & 0\\ 0 & 0 & \frac{E_{c}^{*}}{2(1+\mu_{c})} \end{array}\right]$$

### 2.4. Energy Analysis of CCSs with FGCs

According to the first-order shear deformation theory, the displacement components $\left(u^{\lambda },v^{\lambda },w^{\lambda }\right)$ in the x,θ, and z directions at any point can be denoted by mid-plane displacement components $\left(u_{0}^{\lambda },v_{0}^{\lambda },w_{0}^{\lambda }\right)$ and rotation components $\left(\phi_{x}^{\lambda },\phi_{\theta }^{\lambda }\right)$, which are expressed as follows:

(22) $$u^{\lambda }(x,\theta ,z,t)=u_{0}^{\lambda }(x,\theta ,t)+z\phi_{x}^{\lambda }(x,\theta ,t)$$

(23) $$v^{\lambda }(x,\theta ,z,t)=v_{0}^{\lambda }(x,\theta ,t)+z\phi_{x}^{\lambda }(x,\theta ,t)$$

(24) $$w^{\lambda }(x,\theta ,z,t)=w_{0}^{\lambda }(x,\theta ,t)$$

where $\lambda =\left(c,l\right)$ represent conical and cylindrical shells, and t denotes the time.

The strain components $\left(\varepsilon_{x}^{\lambda },\varepsilon_{\theta }^{\lambda },\varepsilon_{x\theta }^{\lambda },\gamma_{xz}^{\lambda },\gamma_{\theta z}^{\lambda }\right)$ at any point of the FGC-CCS can be determined as follows:

(25) $$\varepsilon_{x}^{\lambda }=\varepsilon_{x0}^{\lambda }+z\kappa_{x}^{\lambda },\;\;\varepsilon_{\theta }^{\lambda }=\varepsilon_{\theta 0}^{\lambda }+z\kappa_{\theta }^{\lambda },\;\;\varepsilon_{x\theta }^{\lambda }=\varepsilon_{x\theta 0}^{\lambda }+z\kappa_{x\theta }^{\lambda },\;\;\gamma_{xz}^{\lambda }=\gamma_{xz0}^{\lambda },\;\;\gamma_{\theta z}^{\lambda }=\gamma_{\theta z0}^{\lambda }$$

where $\varepsilon_{x0}^{\lambda },\;\varepsilon_{\theta 0}^{\lambda }$, $\varepsilon_{x\theta 0}^{\lambda }$ are the membrane strains at the mid-surface, $\gamma_{xz0}^{\lambda }$, $\gamma_{\theta z0}^{\lambda }$ represent the transverse shear strains, and $\kappa_{x}^{\lambda },\;\kappa_{\theta }^{\lambda }$, $\kappa_{x\theta }^{\lambda }$ represent the curvature changes of the structure, respectively.

Then, the strain and displacement relationship for the structure at any point on the mid-plane can start as follows:

(26) $$\varepsilon_{x}^{\lambda }=\frac{\partial u^{\lambda }}{\partial x^{\lambda }},\varepsilon_{\theta }^{\lambda }=\frac{1}{R_{x}}\frac{\partial v^{\lambda }}{\partial \theta^{\lambda }}+\frac{u^{\lambda }}{R_{x}}\frac{\partial R_{x}}{\partial x^{\lambda }}+\frac{w^{\lambda }}{R_{\theta }},\varepsilon_{x\theta }^{\lambda }=\frac{\partial v^{\lambda }}{\partial x^{\lambda }}+\frac{1}{R_{x}}\frac{\partial u^{\lambda }}{\partial \theta^{\lambda }}-\frac{v^{\lambda }}{R_{x}}\frac{R_{x}}{\partial x^{\lambda }}$$

(27) $$\kappa_{x}^{\lambda }=\frac{\partial \phi_{x}^{\lambda }}{\partial x^{\lambda }},\kappa_{\theta }^{\lambda }=\frac{1}{R_{x}}\frac{\partial \phi_{\theta }^{\lambda }}{\partial \theta^{\lambda }}+\frac{\phi_{\theta }^{\lambda }}{R_{x}}\frac{R_{x}}{\partial x^{\lambda }},\kappa_{x\theta }^{\lambda }=\frac{1}{R_{x}}\frac{\partial \phi_{\theta }^{\lambda }}{\partial x^{\lambda }}+\frac{1}{R_{x}}\frac{\partial \phi_{x}^{\lambda }}{\partial \theta^{\lambda }}-\frac{\phi_{\theta }^{\lambda }}{R_{x}}\frac{R_{x}}{\partial x^{\lambda }}$$

(28) $$\gamma_{xz}^{\lambda }=\phi_{x}^{\lambda }+\frac{\partial w^{\lambda }}{\partial x^{\lambda }},\gamma_{\theta z}^{\lambda }=\phi_{\theta }^{\lambda }-\frac{v^{\lambda }}{R_{\theta }}+\frac{1}{R_{x}}\frac{\partial w^{\lambda }}{\partial \theta^{\lambda }}$$

where $\varepsilon_{x}^{\varsigma }$, $\varepsilon_{\theta }^{\varsigma }$, $\varepsilon_{x\theta }^{\varsigma }$ represent the membrane strains, $\kappa_{x}^{\varsigma }$, $\kappa_{\theta }^{\varsigma }$, $\kappa_{x\theta }^{\varsigma }$ are the curvature variables, $\gamma_{xz}^{\varsigma }$, $\gamma_{\theta z}^{\varsigma }$ are the shear strains, and $R_{\theta }$, $R_{x}$ designated the Lamé coefficient and the radius of curvature. For the conical shell, $R_{x}=x\sin\alpha$ and $R_{\theta }=x\tan\alpha$. For the cylindrical shell, $R_{x}=R_{1}$ and $R_{\theta }=R_{1}$.

Therefore, employing the generalized Hooke’s law, the constitutive expression of the combined shells with FGC can be calculated as follows:

(29) $$\left[\begin{array}{l} \sigma_{x}^{\lambda \Gamma }\\ \sigma_{\theta }^{\lambda \Gamma }\\ \tau_{x\theta }^{\lambda \Gamma }\\ \tau_{xz}^{\lambda \Gamma }\\ \tau_{\theta z}^{\lambda \Gamma } \end{array}\right]=\left[\begin{array}{ccccc} {\bar{Q}}_{11}^{\lambda \Gamma } & {\bar{Q}}_{12}^{\lambda \Gamma } & {\bar{Q}}_{16}^{\lambda \Gamma } & 0 & 0\\ {\bar{Q}}_{12}^{\lambda \Gamma } & {\bar{Q}}_{22}^{\lambda \Gamma } & {\bar{Q}}_{26}^{\lambda \Gamma } & 0 & 0\\ {\bar{Q}}_{16}^{\lambda \Gamma } & {\bar{Q}}_{26}^{\lambda \Gamma } & {\bar{Q}}_{66}^{\lambda \Gamma } & 0 & 0\\ 0 & 0 & 0 & {\bar{Q}}_{44}^{\lambda \Gamma } & {\bar{Q}}_{45}^{\lambda \Gamma }\\ 0 & 0 & 0 & {\bar{Q}}_{45}^{\lambda \Gamma } & {\bar{Q}}_{55}^{\lambda \Gamma } \end{array}\right]\left\{\left[\begin{array}{c} \varepsilon_{x}^{\lambda \Gamma }\\ \varepsilon_{\theta }^{\lambda \Gamma }\\ \varepsilon_{x\theta }^{\lambda \Gamma }\\ \gamma_{xz}^{\lambda \Gamma }\\ \gamma_{\theta z}^{\lambda \Gamma } \end{array}\right]-\left[\begin{array}{c} {\bar{\varepsilon }}_{x}^{\lambda \Gamma }\\ {\bar{\varepsilon }}_{\theta }^{\lambda \Gamma }\\ {\bar{\varepsilon }}_{x\theta }^{\lambda \Gamma }\\ {\bar{\gamma }}_{xz}^{\lambda \Gamma }\\ {\bar{\gamma }}_{\theta z}^{\lambda \Gamma } \end{array}\right]\right\}\;\Gamma =s,\;c$$

where $\sigma_{x}^{\lambda }$, $\sigma_{\theta }^{\lambda }$ represent the normal stress along the x and θ directions, respectively, $\tau_{x\theta }^{\lambda }$, $\tau_{xz}^{\lambda }$, $\tau_{\theta z}^{\lambda }$ represent the shear stress values related to the *x−θ*, *x-z,* and *θ-z-directions*, and Γ=s, c represent the CCS and FGC. ${\bar{\varepsilon }}_{\lambda }=({\bar{\varepsilon }}_{x}^{\lambda \Gamma },{\bar{\varepsilon }}_{\theta }^{\lambda \Gamma },{\bar{\varepsilon }}_{x\theta }^{\lambda \Gamma },{\bar{\gamma }}_{xz}^{\lambda \Gamma },{\bar{\gamma }}_{\theta z}^{\lambda \Gamma })$ denote the structure strains in thermal environments. Based on the thermoplastic theory, the above thermal strain can be written as follows:

(30) $$\left[\begin{array}{l} {\bar{\varepsilon }}_{x}^{\lambda s}\\ {\bar{\varepsilon }}_{\theta }^{\lambda s}\\ {\bar{\varepsilon }}_{x\theta }^{\lambda s} \end{array}\right]=\left[\begin{array}{cc} \cos\beta_{k}^{2} & \sin\beta_{k}^{2}\\ \sin\beta_{k}^{2} & \cos\beta_{k}^{2}\\ 2\cos\beta_{k}\sin\beta_{k} & -2\cos\beta_{k}\sin\beta_{k} \end{array}\right]\left[\begin{array}{c} \alpha_{1}\\ \alpha_{2} \end{array}\right]\Delta T$$

(31) $${\bar{\varepsilon }}_{x}^{\lambda c}={\bar{\varepsilon }}_{\theta }^{\lambda c}={\bar{\varepsilon }}_{x\theta }^{\lambda c}=\alpha_{c}\Delta T,\;{\bar{\gamma }}_{xz}^{\lambda \Gamma }={\bar{\gamma }}_{\theta z}^{\lambda \Gamma }=0$$

where $\alpha_{1}$ $\alpha_{2}$, and $\alpha_{c}$ represent the equivalent thermal expansion coefficients of the fiber-reinforced composite substrate and FGC.

Therefore, the internal forces $N_{x}^{\lambda \Gamma }$, $N_{\theta }^{\lambda \Gamma }$, $N_{x\theta }^{\lambda \Gamma }$, bending moments $M_{x}^{\lambda \Gamma }$, $M_{\theta }^{\lambda \Gamma }$, torque $M_{x\theta }^{\lambda \Gamma }$, transverse shear forces $Q_{x}^{\lambda \Gamma }$, $Q_{\theta }^{\lambda \Gamma }$, thermal internal forces $N_{x,T}^{\lambda \Gamma }$, $N_{\theta ,T}^{\lambda \Gamma }$, $N_{x\theta ,T}^{\lambda \Gamma }$, and thermal moments $M_{x,T}^{\lambda \Gamma }$, $M_{\theta ,T}^{\lambda \Gamma }$, $M_{x\theta ,T}^{\lambda \Gamma }$ at any point of the combined shells can be described as follows:

(32) $$\left[\begin{array}{c} N_{x}^{\lambda \Gamma }\\ N_{\theta }^{\lambda \Gamma }\\ N_{x\theta }^{\lambda \Gamma }\\ M_{x}^{\lambda \Gamma }\\ M_{\theta }^{\lambda \Gamma }\\ M_{x\theta }^{\lambda \Gamma }\\ Q_{x}^{\lambda \Gamma }\\ Q_{\theta }^{\lambda \Gamma } \end{array}\right]=\left[\begin{array}{cccccccc} A_{11}^{\lambda \Gamma } & A_{12}^{\lambda \Gamma } & A_{16}^{\lambda \Gamma } & B_{11}^{\lambda \Gamma } & B_{12}^{\lambda \Gamma } & B_{16}^{\lambda \Gamma } & 0 & 0\\ A_{12}^{\lambda \Gamma } & A_{22}^{\lambda \Gamma } & A_{26}^{\lambda \Gamma } & B_{12}^{\lambda \Gamma } & B_{22}^{\lambda \Gamma } & B_{26}^{\lambda \Gamma } & 0 & 0\\ A_{16}^{\lambda \Gamma } & A_{26}^{\lambda \Gamma } & A_{66}^{\lambda \Gamma } & B_{16}^{\lambda \Gamma } & B_{26}^{\lambda \Gamma } & B_{66}^{\lambda \Gamma } & 0 & 0\\ B_{11}^{\lambda \Gamma } & B_{12}^{\lambda \Gamma } & B_{16}^{\lambda \Gamma } & D_{11}^{\lambda \Gamma } & D_{12}^{\lambda \Gamma } & D_{16}^{\lambda \Gamma } & 0 & 0\\ B_{12}^{\lambda \Gamma } & B_{22}^{\lambda \Gamma } & B_{26}^{\lambda \Gamma } & D_{12}^{\lambda \Gamma } & D_{22}^{\lambda \Gamma } & D_{26}^{\lambda \Gamma } & 0 & 0\\ B_{16}^{\lambda \Gamma } & B_{26}^{\lambda \Gamma } & B_{66}^{\lambda \Gamma } & D_{16}^{\lambda \Gamma } & D_{26}^{\lambda \Gamma } & D_{66}^{\lambda \Gamma } & 0 & 0\\ 0 & 0 & 0 & 0 & 0 & 0 & \bar{\kappa }A_{45}^{\lambda \Gamma } & \bar{\kappa }A_{55}^{\lambda \Gamma }\\ 0 & 0 & 0 & 0 & 0 & 0 & \bar{\kappa }A_{44}^{\lambda \Gamma } & \bar{\kappa }A_{45}^{\lambda \Gamma } \end{array}\right]\left[\begin{array}{c} \varepsilon_{x}^{\lambda \Gamma }\\ \varepsilon_{\theta }^{\lambda \Gamma }\\ \varepsilon_{x\theta }^{\lambda \Gamma }\\ \kappa_{x}^{\lambda \Gamma }\\ \kappa_{\theta }^{\lambda \Gamma }\\ \kappa_{x\theta }^{\lambda \Gamma }\\ \gamma_{xz}^{\lambda \Gamma }\\ \gamma_{\theta z}^{\lambda \Gamma } \end{array}\right]$$

(33) $$\left[\begin{array}{c} N_{x,T}^{\lambda \Gamma }\\ N_{\theta ,T}^{\lambda \Gamma }\\ N_{x\theta ,T}^{\lambda \Gamma } \end{array}\right]=\int_{h_{\Gamma }}\left[\begin{array}{ccc} {\bar{Q}}_{11}^{\lambda \Gamma } & {\bar{Q}}_{12}^{\lambda \Gamma } & {\bar{Q}}_{16}^{\lambda \Gamma }\\ {\bar{Q}}_{12}^{\lambda \Gamma } & {\bar{Q}}_{22}^{\lambda \Gamma } & {\bar{Q}}_{26}^{\lambda \Gamma }\\ {\bar{Q}}_{16}^{\lambda \Gamma } & {\bar{Q}}_{26}^{\lambda \Gamma } & {\bar{Q}}_{66}^{\lambda \Gamma } \end{array}\right]\left[\begin{array}{c} {\bar{\varepsilon }}_{x}^{\lambda \Gamma }\\ {\bar{\varepsilon }}_{\theta }^{\lambda \Gamma }\\ {\bar{\varepsilon }}_{x\theta }^{\lambda \Gamma } \end{array}\right]\mathrm{dz},\;\left[\begin{array}{c} M_{x,T}^{\lambda \Gamma }\\ M_{\theta ,T}^{\lambda \Gamma }\\ M_{x\theta ,T}^{\lambda \Gamma } \end{array}\right]=\int_{h_{\Gamma }}\left[\begin{array}{ccc} {\bar{Q}}_{11}^{\lambda \Gamma } & {\bar{Q}}_{12}^{\lambda \Gamma } & {\bar{Q}}_{16}^{\lambda \Gamma }\\ {\bar{Q}}_{12}^{\lambda \Gamma } & {\bar{Q}}_{22}^{\lambda \Gamma } & {\bar{Q}}_{26}^{\lambda \Gamma }\\ {\bar{Q}}_{16}^{\lambda \Gamma } & {\bar{Q}}_{26}^{\lambda \Gamma } & {\bar{Q}}_{66}^{\lambda \Gamma } \end{array}\right]\left[\begin{array}{c} {\bar{\varepsilon }}_{x}^{\lambda \Gamma }\\ {\bar{\varepsilon }}_{\theta }^{\lambda \Gamma }\\ {\bar{\varepsilon }}_{x\theta }^{\lambda \Gamma } \end{array}\right]\mathrm{zdz}$$

where $\bar{\kappa }$ is the shear correction factor; according to the literature [51], it can be determined as 5/6. Moreover, $A_{ij}^{\lambda \Gamma }$, $B_{ij}^{\lambda \Gamma }$, and $D_{ij}^{\lambda \Gamma }(i,j=1,2,4,5,6)$ represent the tension, tension-bending coupling, and bending stiffness coefficients of the shell with coatings, and can be obtained by the following formula:

(34) $$\left(A_{ij}^{\lambda s},B_{ij}^{\lambda s},D_{ij}^{\lambda s}\right)=\int_{-h}^{h_{s}-h}{\bar{Q}}_{ij}^{\left(s\right)}\;\left(1,z,z^{2}\right)\mathrm{dz},\;\left(A_{ij}^{\lambda c},B_{ij}^{\lambda c},D_{ij}^{\lambda c}\right)=\int_{h_{s}-h}^{h_{s}-h+h_{c}}{\bar{Q}}_{ij}^{\left(c\right)}\;\left(1,z,z^{2}\right)\mathrm{dz}$$

The kinetic energy expression of the combined shells with FGC is assumed to be as follows:

(35) $$T_{K}^{\lambda }=\frac{1}{2}\int_{\theta }\int_{x}\left\{\begin{array}{l} I_{0}^{\lambda }\left[{\left(\frac{\partial u_{0}^{\lambda }}{\partial t}\right)}^{2}+{\left(\frac{\partial v_{0}^{\lambda }}{\partial t}\right)}^{2}+{\left(\frac{\partial w_{0}^{\lambda }}{\partial t}\right)}^{2}\right]+2I_{1}^{\lambda }\left[\left(\frac{\partial u_{0}^{\lambda }}{\partial t}\frac{\partial \phi_{x}^{\lambda }}{\partial t}+\frac{\partial v_{0}^{\lambda }}{\partial t}\frac{\partial \phi_{\theta }^{\lambda }}{\partial t}\right)\right]\\ +I_{2}^{\lambda }\left[{\left(\frac{\partial \phi_{x}^{\lambda }}{\partial t}\right)}^{2}+{\left(\frac{\partial \phi_{\theta }^{\lambda }}{\partial t}\right)}^{2}\right] \end{array}\right\}R_{x}dxd\theta$$

where $I_{0}^{\lambda }$, $I_{1}^{\lambda }$, and $I_{2}^{\lambda }$ represent the inertia terms of the combined shell and functionally graded coating, and their expressions are as follows:

(36) $$\left[I_{0}^{s},I_{1}^{s},I_{2}^{s}\right]=\int_{-h}^{h_{s}-h}\rho_{s}\left[1,z,z^{2}\right]\mathrm{dz},\;\left[I_{0}^{c},I_{1}^{c},I_{2}^{c}\right]=\int_{h_{s}-h}^{h_{s}-h+h_{c}}\rho_{c}\left[1,z,z^{2}\right]\mathrm{dz}$$

Then, the strain energy $U_{s}^{\lambda \Gamma }$ of the structure can be expressed as follows:

(37) $$U_{s}^{\lambda \Gamma }=\frac{1}{2}\iint_{A}\left(N_{x}^{\lambda \Gamma }\varepsilon_{x}^{\lambda \Gamma }+N_{\theta }^{\lambda \Gamma }\varepsilon_{\theta }^{\lambda \Gamma }+N_{x\theta }^{\lambda \Gamma }\varepsilon_{x\theta }^{\lambda \Gamma }+Q_{x}^{\lambda \Gamma }\gamma_{xz}^{\lambda \Gamma }+M_{x}^{\lambda \Gamma }\kappa_{x}^{\lambda \Gamma }+M_{\theta }^{\lambda \Gamma }\kappa_{\theta }^{\lambda \Gamma }+M_{x\theta }^{\lambda \Gamma }\kappa_{x\theta }^{\lambda \Gamma }+Q_{\theta }^{\lambda \Gamma }\gamma_{\theta z}^{\lambda \Gamma }\right)R_{x}dA$$

Considering the temperature field, the thermal potential energy generated by the structure can be stated as follows:

(38) $$U_{T}^{\lambda \Gamma }=\frac{1}{2}\iint_{A}\left\{N_{x,T}^{\lambda \Gamma }{\left(\frac{\partial w_{0}^{\varsigma }}{\partial x}\right)}^{2}+N_{\theta ,T}^{\lambda \Gamma }{\left(\frac{\partial w_{0}^{\varsigma }}{R_{x}\partial \theta }\right)}^{2}+2N_{x\theta ,T}^{\lambda \Gamma }\frac{\partial w_{0}^{\varsigma }}{\partial x}\frac{\partial w_{0}^{\varsigma }}{R_{x}\partial \theta }\right\}R_{x}dA$$

The potential energy generated by the boundary springs at both ends of the composite shells with FGC can be described as follows:

(39) $$U_{\mathrm{sp}}^{\Gamma }=\frac{1}{2}\left\{\begin{array}{l} {\int_{0}^{2\pi }\left[k_{1}{\left(u_{0}^{c}\right)}^{2}+k_{2}{\left(v_{0}^{c}\right)}^{2}+k_{3}{\left(w_{0}^{c}\right)}^{2}+k_{4}{\left(\phi_{x}^{c}\right)}^{2}+k_{5}{\left(\phi_{\theta }^{c}\right)}^{2}\right]}_{\left(x_{1}=0\right)}R_{x}d\theta_{1}+\\ {\int_{0}^{2\pi }\left[k_{6}{\left(u_{0}^{l}\right)}^{2}+k_{7}{\left(v_{0}^{l}\right)}^{2}+k_{8}{\left(w_{0}^{l}\right)}^{2}+k_{9}{\left(\phi_{x}^{l}\right)}^{2}+k_{10}{\left(\phi_{\theta }^{l}\right)}^{2}\right]}_{\left(x_{2}=L_{2}\right)}R_{x}d\theta_{2} \end{array}\right\}$$

The potential energy generated in the connecting spring has the following expression:

(40) $$U_{\mathrm{con}}^{\Gamma }=\frac{1}{2}\int_{0}^{2\pi }\left\{\begin{array}{c} k_{u}{\left(u_{0}^{l}-u_{0}^{c}\cos\alpha +w_{0}^{c}\sin\alpha \right)}^{2}+k_{v}{\left(v_{0}^{l}-v_{0}^{c}\right)}^{2}+\\ k_{w}{\left(w_{0}^{l}-u_{0}^{c}\sin\alpha -w_{0}^{c}\cos\alpha \right)}^{2}+k_{x}{\left(\phi_{x}^{l}-\phi_{x}^{c}\right)}^{2}+k_{\theta }{\left(\phi_{\theta }^{l}-\phi_{\theta }^{c}\right)}^{2} \end{array}\right\}R_{x}d\theta$$

### 2.5. Displacement Function Assumptions and Solutions

In this study, the Jacobi polynomial is used to assume the mid-surface displacement function. The Jacobi polynomial can be derived using the following recursive method, within the range $\varphi \in \left[-1,1\right]$ [52]:

(41) $$P_{0}^{\left(a,b\right)}(\varphi )=1,P_{1}^{\left(a,b\right)}(\varphi )=\frac{a+b+2}{2}\varphi -\frac{a-b}{2}$$

(42) $$\begin{array}{c} P_{m}^{\left(a,b\right)}(\varphi )=\frac{(a+b+2m-1)\left[a^{2}-b^{2}+\varphi (a+b+2m)(a+b+2m-2)\right]}{2m(a+b+m)(a+b+2m-2)}P_{m-1}^{\left(a,b\right)}(\varphi )\\ -\frac{\left(a+m-1)(b+m-1)(a+b+2m\right)}{m(a+b+m)(a+b+2m-2)}P_{m-2}^{\left(a,b\right)}(\varphi ) \end{array}$$

where a,b>1 are the Jacobi polynomial coefficients.

The displacement field in the neutral plane of the structure is defined as follows:

(43) $$u_{0}^{\lambda }(x,\theta ,t)=\sum_{m=0}^{M}A_{mn}^{\lambda }P_{m}^{\left(a,b\right)}(\varphi )\cos(n\theta )e^{i\omega t},\;\;v_{0}^{\lambda }(x,\theta ,t)=\sum_{m=0}^{M}B_{mn}^{\lambda }P_{m}^{\left(a,b\right)}(\varphi )\sin(n\theta )e^{i\omega t}$$

(44) $$w_{0}^{\lambda }(x,\theta ,t)=\sum_{m=0}^{M}C_{mn}^{\lambda }P_{m}^{\left(a,b\right)}(\varphi )\cos(n\theta )e^{i\omega t},\;\;\phi_{x}^{\lambda }(x,\theta ,t)=\sum_{m=0}^{M}D_{mn}^{\lambda }P_{m}^{\left(a,b\right)}(\varphi )\cos(n\theta )e^{i\omega t}$$

(45) $$\phi_{\theta }^{\lambda }(x,\theta ,t)=\sum_{m=0}^{M}E_{mn}^{\lambda }P_{m}^{\left(a,b\right)}(\varphi )\cos(n\theta )e^{i\omega t}$$

where $A_{mn}^{\lambda }$, $B_{mn}^{\lambda }$, $C_{mn}^{\lambda }$, $D_{mn}^{\lambda }$, and $E_{mn}^{\lambda }$ represent the Ritz coefficients, *m* and *n* represent the axial and circumferential half-wave numbers, ω is the circular frequency, and *M* is the axial truncation number of the Jacobi polynomial.

The Lagrange energy function ∏ of the CCSs with FGCs is expressed as follows:

(46) $$\prod =T_{K}^{\lambda }-U_{s}^{\lambda \Gamma }-U_{T}^{\lambda \Gamma }-U_{\mathrm{sp}}^{\Gamma }-U_{\mathrm{con}}^{\Gamma }$$

According to the Rayleigh–Ritz method, by taking the partial derivative of the Ritz coefficient in the Lagrange energy function constructed by Equation (31), the following can be obtained:

(47) $$\frac{\partial \prod }{\partial \vartheta }=0\;(\vartheta =A_{mn}^{\lambda },B_{mn}^{\lambda },C_{mn}^{\lambda },D_{mn}^{\lambda },E_{mn}^{\lambda })$$

Consequently, the following matrix representation can be derived from the aforementioned formula:

(48) $$(K_{*}-\omega^{2}M)q=0$$

where $K_{*}=K+iC$ and M represent the complex stiffness and complex mass matrix of the structure, K and C are the stiffness and damping matrix, and q is the vibration mode vector.

To solve the fundamental frequency of the combined shells with FGC, set C = 0. Using this condition, the following formula can be obtained:

(49) $$(K-\omega^{2}M)q=0$$

Furthermore, assuming that the shell with a coating structure is excited by the fundamental harmonic $F_{b}$ in the z-direction, the analysis can begin as follows:

(50) $$F_{b}=f_{b}e^{i\omega_{*}t}$$

where $f_{b}$ and $\omega_{*}$ represent the excitation amplitude and angular frequency, respectively.

Moreover, using the modal strain energy approach, the damping ratio of the *r*-th mode can be represented as follows:

(51) $$\zeta_{r}=\frac{\xi_{r}}{2\pi }=\frac{1}{4\pi }\frac{q_{r}^{T}Cq_{r}}{q_{r}^{T}Kq_{r}}$$

where $q_{r}$ is the *r*-th vibration mode vector.

Then, to address the forced vibration response, the mass matrix M, stiffness matrix K, and damping matrix C are regularized to obtain the regularized mass matrix $\bar{M}$, stiffness matrix $\bar{K}$, and damping matrix $\bar{C}$. The regularization process are as follows:

(52) $$\bar{M}=\mathrm{diag}\left[{\left(q_{r}^{T}\right)}^{-1}M_{r}q_{r}^{-1}\right]$$

(53) $$\bar{K}=\mathrm{diag}\left[{\left(q_{r}^{T}\right)}^{-1}M_{r}\omega_{r}^{2}q_{r}^{-1}\right]$$

(54) $$\bar{C}=\mathrm{diag}\left[{\left(q_{r}^{T}\right)}^{-1}i2\omega_{r}^{2}M_{r}\xi q_{r}^{-1}\right]$$

where $\omega_{r}$ represents the *r*-th order natural circular frequency, and $M_{r}$ is the *r*-th order modal mass, which can be defined by the following formula:

(55) $$M_{r}=q_{r}^{T}Mq_{r}$$

In addition, by arranging Equations (52)–(55), the expression of the complex frequency response function $H\left(\omega \right)$ of the CCSs with FGCs can be written as follows:

(56) $$H\left(\omega \right)={\left[\bar{K}+i\bar{C}-\omega_{*}^{2}\bar{M}\right]}^{-1}=\sum_{r=1}^{N_{m}}\frac{M_{r}q_{r}^{T}q_{r}}{\omega_{r}\left(1+i2\xi_{r}\right)-\omega_{*}^{2}}$$

where $N_{m}$ is the maximum modal order of the structure.

By adopting the mode superposition method [53], the steady-state response amplitude function $D_{s}\left(\omega \right)$ of the structure at the response point $R_{p}\left(x,\theta ,z\right)$ is derived as follows:

(57) $$D_{s}\left(\omega \right)=\left|H\left(\omega \right)\right|{\bar{F}}_{b}=\sum_{r=1}^{N_{m}}\frac{W_{r0}W_{r1}\left(x,\theta ,z\right)}{\sqrt{\left(1-\omega_{*}^{2}/\omega_{r}^{2}\right)+{\left(2\xi_{r}\right)}^{2}}}\frac{f_{b}}{K_{r}}$$

(58) $$W_{r0}=\int_{0}^{2\pi }\int_{0}^{L}\left[Sub\left(w_{0}^{\lambda },q_{r}\right)\right]I_{0}\cos\left(n\theta \right)R_{x}dxd\theta$$

(59) $$W_{r1}=\left(x,\theta ,z\right)=Sub\left[Sub\left(w_{0}^{\lambda },q_{r}\right),R_{p}\right]$$

where ${\bar{F}}_{b}$ is the equivalent force load of the basic excitation, $W_{r0}$ and $W_{r1}$ represent the *r*-th displacement function associated with the excitation direction and the response point [54], $Sub\left(a,b\right)$ means to bring the value of b into the formula of a, and $K_{r}$ represents the *r*-th modal stiffness; the expression is as follows:

(60) $$K_{r}=q_{r}^{T}Kq_{r}$$

Finally, by substituting the fundamental frequency into Equation (56), the steady-state resonant response amplitude can be obtained.

## 3. Experimental Test

The vibrational properties of coated and uncoated CCSs in thermal environments are thoroughly examined in this section, and the experimental data obtained from these measurements are subsequently compared to theoretical predictions, thereby offering powerful validation for the present model.

### 3.1. Specimen Preparation

Two uncoated specimens made of polylactic acid resin (PLA) and continuous carbon fiber are manufactured by continuous 3D printing technology. Each specimen has 20 layers, with uniform fiber layer thicknesses and a [0°/90°]_10_ layout pattern. Furthermore, the relevant material parameters of the two uncoated specimens are given by the manufacturer and are detailed in Table 2. Subsequently, a layer of functionally graded material is coated on the surface of one of the specimens using atomization deposition technology, thereby producing a conical-cylindrical shell specimen with a functionally graded coating. The functionally graded coating consisted of six mixed layers of components A and B, each with a thickness of 0.1 mm. In this advanced coating, component A is composed of ceramic material, and component B is an organic solvent that works as a bond, to which high-temperature resistant fillers and functional inorganic fillers have been added.

**Figure 3:**
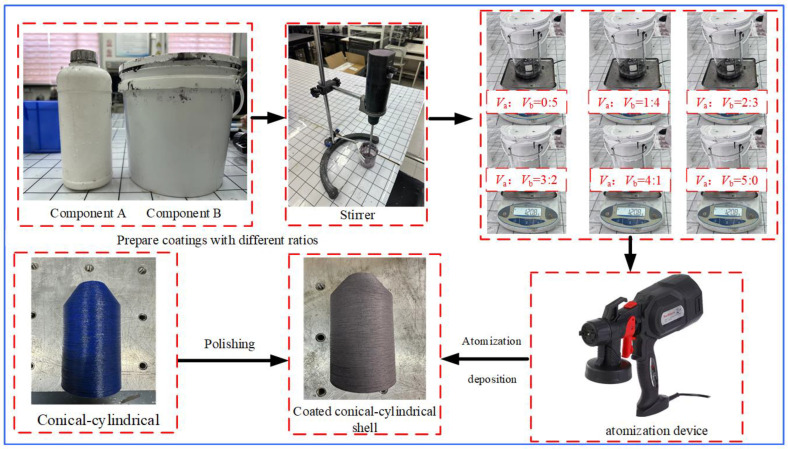
Fabrication of conical-cylindrical shells with functionally graded coatings.

The following steps are primarily involved in the multilayer coating preparation process: (1) Prepare mixed slurries in different proportions in advance. Add components A and B into six beakers, according to a certain proportion, stir with a stirrer at room temperature until all the precipitates are evenly dispersed, and then filter through filter cloth; it should be emphasized that complete mixing of the two components is frequently difficult to achieve in practice, as stated in the literature [55]. However, throughout the experimental process, we assume that they are perfectly combined. (2) Polish the specimen surface. Use sandpaper to evenly polish the specimen surface to increase its roughness, thereby increasing the interface bonding strength between the coating and the specimen surface. (3) Atomization deposition. Pour the prepared coating slurry into the atomization device and place the device at an appropriate height directly above the specimen. Turn on the atomizer to allow the coating slurry to be evenly distributed on the surface of the specimen to form a deposition, to guarantee consistent coating, the specimen is positioned on a slowly turning support. The coating thickness is measured using a micrometer and the thickness of each layer can be controlled by the amount of components A and B sprayed. (4) Coating curing. To cure the coating on the surface of the specimen, heating can be applied to accelerate the curing process, or it can be allowed to cure naturally at room temperature. After the curing is complete, the first layer of the coating is ready. By repeating the steps mentioned above, a specimen with six layers of functionally graded coatings can be achieved. Figure 3 illustrates the preparation process for the CCSs with FGC.

### 3.2. Test System and Experimental Method

**Figure 4:**
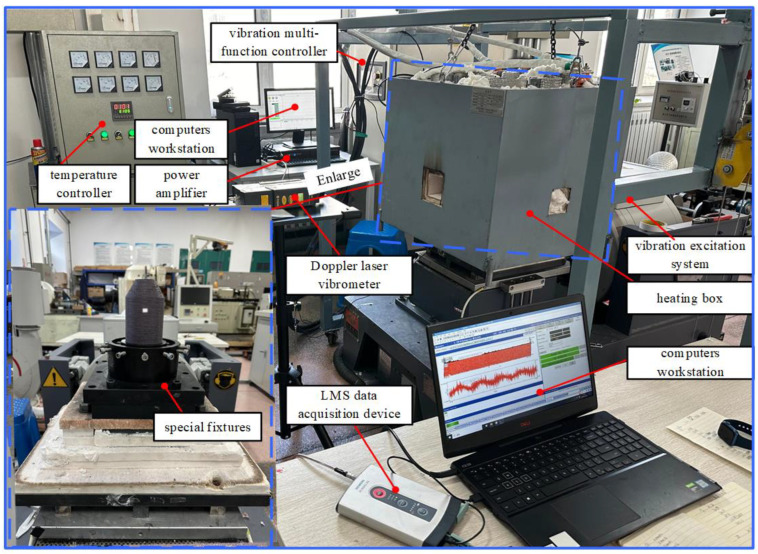
A thermal vibration test system for uncoated and coated conical-cylindrical shells.

A thermal vibration test system for coated and uncoated CCS specimens is built, as shown in Figure 4. The system is mainly composed of a vibration excitation system, a vibration multi-function controller, a power amplifier, a thermocouple sensor, a heating box, a temperature controller, a Doppler laser vibrometer, a set of special fixtures, an LMS data acquisition device and two computers workstation. Before the vibration test begins, the special fixture is tightened on the vibration table, and one end of the conical-cylindrical shell is fixed to the special fixture using the torque wrench to simulate the boundary condition of a free section fixed at one end in actual applications, ensuring that the excitation provided by the excitation system and power amplifier can be efficiently transmitted to the specimen.

Simultaneously, the hot box is slowly lowered onto the specimens via the slide until the bottom of the hot box is entirely in touch with the insulation base, and the specimens are heated by the heating box to achieve a consistent thermal environment at a preset temperature. Moreover, the thermocouple sensor is installed in the heating box and measures and monitors changes in environmental temperature in real time. Meanwhile, the vibration signal of the specimen is measured by the Doppler laser vibrometer, and the laser vibration measurement point is located at $\left(L_{\mathrm{cy}}/2\pi ,\pi /3,R_{\mathrm{cy}}+h\right)$. These signals are recorded by an Siemens LMS data-collecting device purchased from Munich, Germany and the relevant data are processed and analyzed by the 2021 version LMS TestLab software installed on the laptop.

The required test temperature is set by adjusting the temperature controller of the hot box. Then, the temperature is quickly heated to the expected temperature by heating the molybdenum rod. After the temperature stabilizes, a series of frequency sweep experiments are started. The half-power bandwidth method [56] and peak recognition method [57] are utilized to extract the appropriate natural frequencies and damping results in various modes from the test sweep data. Then, the required test temperature is set by adjusting the hot box temperature controller, and a series of frequency sweep experiments are started when the temperature stabilizes. Subsequently, a series of fixed-frequency experiments are performed to examine the vibration response of the specimen when the vibration suppression capability of the coating is taken into account. In addition, the modal vibration shape can be obtained through a hammer test. To record the reaction data, the accelerometer is positioned at the response point $R_{p}$ and the impact is applied multiple times at different positions to record the response data. The modal vibration shape is then determined by the modal parameter identification method. The important parameters of the experiment process are as follows: (1) frequency resolution: 0.16; (2) frequency range: 100–1600 Hz; (3) experimental temperature: 50 °C and 100 °C; (4) excitation amplitude: 2 g.

### 3.3. Numerical Verification and Analysis

To verify the accuracy of the proposed theoretical model of FGC-combined shells in thermal environments, thermal vibration tests are carried out on uncoated and coated combined shell specimens (named S-1 and S-2), respectively. The geometric parameters and material parameters used are shown in Table 2.

**Figure 5:**
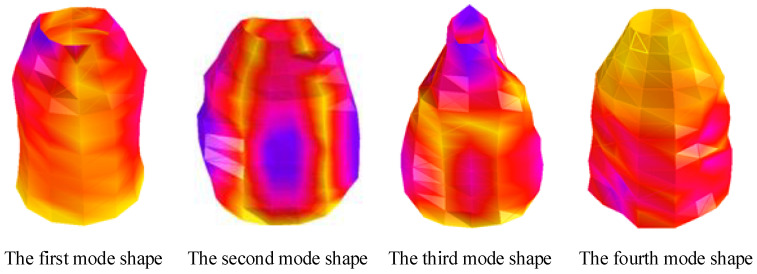
The first four modal vibration shapes obtained through experiments.

**Figure 6:**
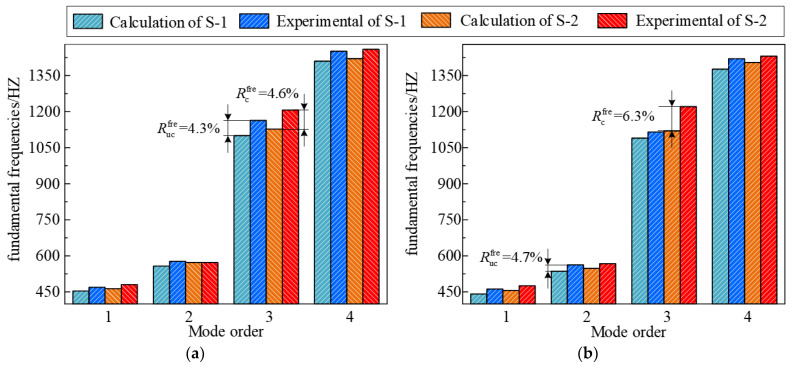
The first four fundamental frequencies derived from calculations and experimental tests of specimens with the environmental temperature of 50 °C and 100 °C; (**a**) 50 °C; (**b**) 100 °C.

Figure 5 shows the first four modal vibration shapes of the structure obtained through experiments, clearly showing the mode shape corresponding to each fundamental frequency. Furthermore, Figure 6 compares the theoretical calculations and experimental test findings for the first four orders of natural frequencies of S-1 and 2 at 50 °C and 100 °C, respectively. Figure 6 illustrates that under the conditions of 50 °C and 100 °C, the maximum errors, $R_{\mathrm{uc}}^{\mathrm{fre}}$, of the natural frequency value of S-1 obtained through theoretical calculations and experimental testing are 4.3% and 4.7%, while the maximum errors, $R_{c}^{\mathrm{fre}}$, of S-2 are 4.6% and 6.3%, respectively. The reason for the error between the third-order modal theoretical calculation and the experimental test could be that the current structure is connected with a certain mode of the fixture or another structure. On the other hand, the deformation characteristics of the third-order mode may be more sensitive to the characteristics of the material and the geometric shape of the structure. Simultaneously, the equipment noise or frequency response characteristics also may lead to this phenomenon. However, the calculation errors are within reasonable limits, which indicates that the current model can reliably predict the free vibration properties of CCSs with FGCs in thermal environments.

**Figure 7:**
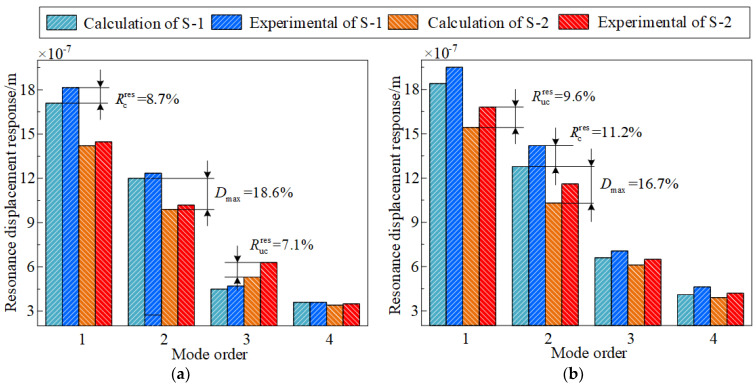
The first four resonance displacement responses derived from calculations and experimental tests of specimens with the environmental temperature of 50 °C and 100 °C, (**a**) 50 °C, (**b**) 100 °C.

Furthermore, to further assess the vibration suppression ability of the FGC, the first four orders of displacement response of S-1 and S-2 at 50 °C and 100 °C are investigated. The first four orders of resonance displacement responses of S-1 and S-2 obtained through theoretical calculation and experimental testing under the conditions of 2 g basic excitation are compared, as shown in Figure 7. It can be observed that at a temperature of 50 °C, the resonance displacement response error between the theoretical calculations and experimental measurements for S-1 and 2 is 7.1% and 8.7%, respectively. At 100 °C, the maximum errors for S-1 and 2 are 9.6% and 11.2%. In addition, Figure 7 indicates that the vibration response of the combined shells decreases by 18.6% and 16.7%, respectively, at two temperatures after the functionally graded coating is applied. Meanwhile, a comparison of Figure 7a,b reveals a decreasing trend in the resonance displacement response of the specimens as the temperature increases from 50 °C to 100 °C. This phenomenon can be explained by the reduction in the elastic modulus of the material with rising temperature, which eventually causes the stiffness of the structure to diminish, resulting in a lowered resonance response. Thus, it can be concluded that the application of FGC can effectively minimize the vibration response of the CCSs, and the currently presented model can forecast the vibration behavior of the currently examined structure in a thermal environment with reliability. However, the sources of errors in theoretical calculations and experimental tests need to be analyzed, which are likely caused by the following: (1) friction at the interface between the substrate and the coating is not considered; (2) the manufacturing error of fiber-reinforced composite shells is ignored; (3) in theoretical modeling, assume that the material behaves linearly and neglect the nonlinear properties of the structure.

## 4. Parametric Analysis

Employing the verified model, the effect of some critical factors on the vibration properties of the CCSs with FGCs in a thermal environment is investigated. Unless otherwise stated, the structural parameters from Section 3.3 are used in the calculations below, with the same excitation amplitude. It is worth noting that normalization is used to derive the resonant displacement response and fundamental frequency.

### 4.1. Effect of Coating Gradient Index on Vibration Property

**Figure 8:**
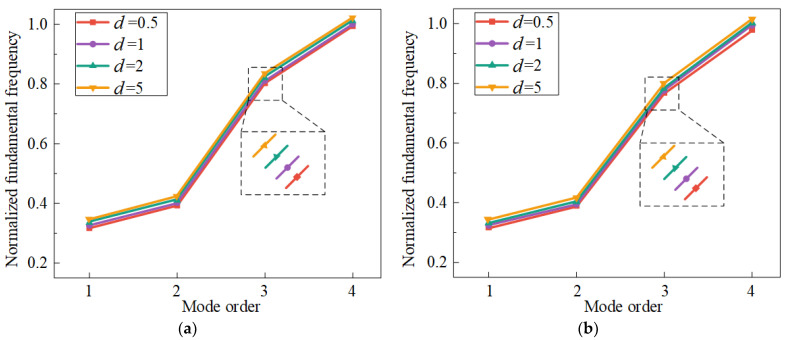
Effects of the functionally graded index on the first four fundamental frequencies of the structure with the environmental temperature of 50 °C and 100 °C, (**a**) 50 °C, (**b**) 100 °C.

**Figure 9:**
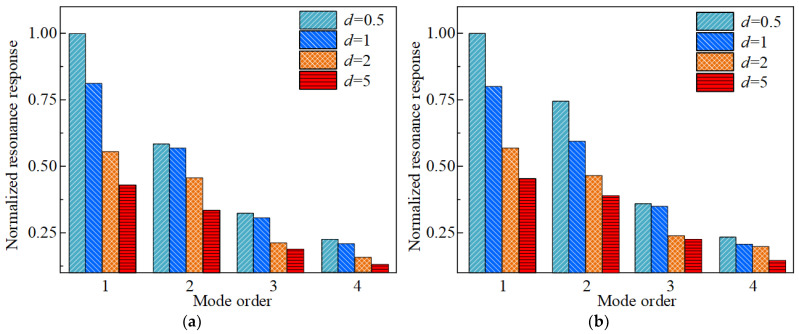
Effects of the functionally graded index on the first four resonance displacement response of the structure with the environmental temperature of 50 °C and 100 °C, (**a**) 50 °C, (**b**) 100 °C.

Firstly, the impact of the functionally graded index on the structural vibration properties is analyzed, as shown in Figure 8 and Figure 9. The normalized fundamental frequencies all exhibit an upward trend at temperatures of 50 °C and 100 °C when the functionally graded index increases. This phenomenon is primarily caused by the bending stiffness, tensile stiffness, and tensile-bending coupling stiffness in Equation (8) increase as the gradient index increases, which increases the total mass and stiffness of the structure. Moreover, Figure 9 depicts the impact of different functionally graded indices on the resonance displacement response. It is evident that the resonance response tends to decline with increasing gradient index. The research above demonstrates that the increase of functionally graded index can enhance the vibration resistance of the CCSs. Therefore, selecting the largest possible functional gradient index, according to engineering design requirements, is recommended.

### 4.2. Effect of FGC Thickness Proportion on Vibration Property

**Figure 10:**
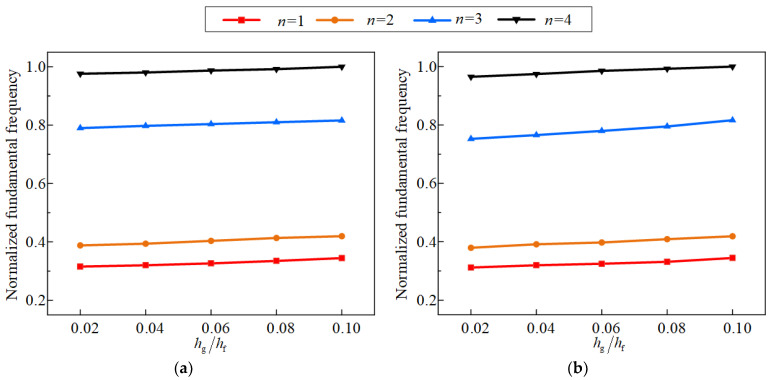
Effects of different thickness ratios on the first four fundamental frequencies of the CCSs with FGCs with the environmental temperature of 50 °C and 100 °C, (**a**) 50 °C, (**b**) 100 °C.

**Figure 11:**
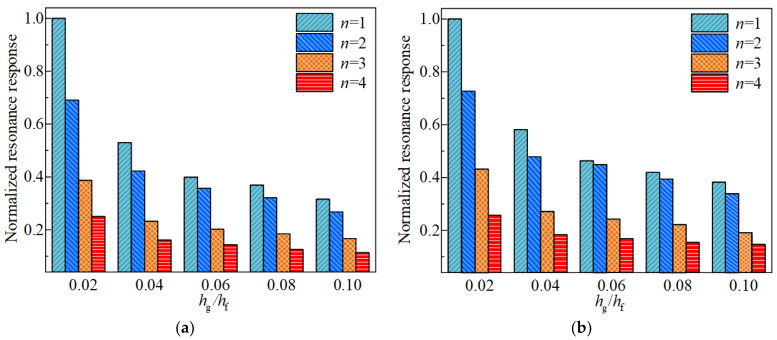
Effects of different thickness ratios on the first four resonance responses of the CCSs with FGCs, with environmental temperatures of 50 °C and 100 °C, (**a**) 50 °C, (**b**) 100 °C.

Subsequently, the influence of the FGC thickness to CCSs shell thickness ratio on the structural vibration property under conditions of 50 °C and 100 °C are investigated, in order to provide a reference for practical engineering, the thickness ratio is selected in the range of 0.02–0.1 for analysis, as indicated in Figure 10 and Figure 11. Assuming the overall thickness of the structure remains constant, it can be observed that as the thickness ratio $h_{g}/h_{f}$ increases from 0.02 to 0.1, the fundamental frequency of the structure exhibits an increasing trend, while the resonance displacement response shows an opposite trend. This phenomenon is due to the stiffness of the structure increases with the rising $h_{g}/h_{f}$. Therefore, to improve the damping ability of the FGC, a higher thickness ratio is advised.

### 4.3. Effect of Young’s Modulus Ratio between FGC and CCSs

**Figure 12:**
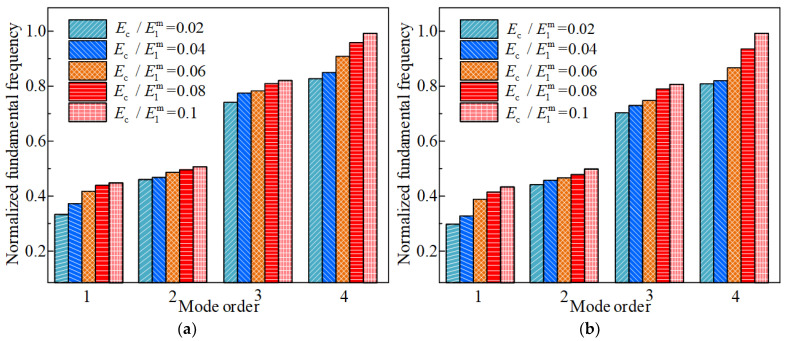
Effects of Young’s modulus ratio between FGCs and CCSs on the first four fundamental frequencies of the CCSs with FGCs with environmental temperatures of 50 °C and 100 °C, (**a**) 50 °C, (**b**) 100 °C.

**Figure 13:**
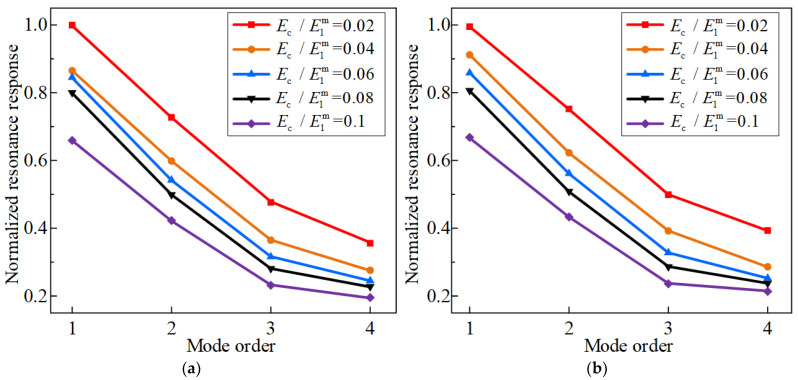
Effects of Young’s modulus ratio between FGCs and CCSs on the first four resonance response of the CCSs with FGCs with the environmental temperature of 50 °C and 100 °C, (**a**) 50 °C, (**b**) 100 °C.

Finally, the effect of Young’s modulus ratio of the FGC to the CCSs on the vibration property is studied. Figure 12 and Figure 13 describe that at 50 °C and 100 °C, the normalized fundamental frequency of the structure increases with the increase of $E_{c}/E_{1}^{m}$, while the resonant displacement response decreases with the increase of the $E_{c}/E_{1}^{m}$. As the $E_{c}/E_{1}^{m}$ increases, the overall strain energy of the coated shells also increases, increasing stiffness. From the above analysis, it can be concluded that selecting an appropriately large Young’s modulus ratio has a positive effect on the vibration suppression of the CCSs.

## 5. Conclusions

This study focuses on the vibration characteristics of the structure at specific temperatures of 50 °C and 100 °C. In this research, a theoretical model for analyzing the vibration behaviors of conical-cylindrical shells with FGC in a temperature environment is established. Theoretical and experimental research is being conducted to investigate their vibrational characteristics under different temperature conditions. Some significant findings are drawn from the computations and experimental data, as follows:

(1) The current model can reliably predict the vibration behavior of coated and uncoated specimens in thermal environments, and the vibration features of the structure are affected by the temperature. As the temperature rises, the fundamental frequency of the CCS with FGC tends to decrease, but the resonant response steadily increases.
(2) Experimental and theoretical studies show that the specimens with functionally graded coatings have a smaller resonant response than those without coatings. Therefore, it can be concluded that FGC contributes to the ability of the structure to suppress vibration.
(3) The dynamic behavior of the CCS with FGC is significantly impacted by the material parameters of the functionally graded coating, such as the functionally graded index, thickness, and Young’s modulus. Therefore, reasonably selecting the above parameters to enhance the anti-vibration capabilities of the structure is recommended.

## Figures and Tables

**Table 1:** Nomenclature of conical-cylindrical shells and functionally graded coating.

Type	Young’s Modulus	Shear Modulus	Poisson’s Ratio	Density
The fiber-reinforced materials	$E_{1}^{f},E_{2}^{f}$	$G_{12}^{f},G_{13}^{f},G_{23}^{f}$	$\mu_{12}^{f},\mu_{21}^{f}$	$\rho_{f}$
The polymer matrix	$E_{p}$	$G_{p}$	$\mu_{p}$	$\rho_{p}$
Coating component A	$E_{a}$	$G_{a}$	$\mu_{a}$	$\rho_{a}$
Coating component B	$E_{b}$	$G_{b}$	$\mu_{b}$	$\rho_{b}$

**Table 2:** Geometric and material parameters of the CCS substrate and FGC.

Type	Geometry and Material Parameters
CCSs FGC	$E_{1}^{m}=15E_{2}^{m}$, $E_{2}^{m}=4.2\;GPa$, $G_{12}^{m}=0.6E_{2}^{m}$, $G_{13}^{m}=G_{12}^{m}$, $G_{23}^{m}=0.5E_{2}^{m}$, $\mu_{12}=0.25$, $\beta_{k}={\left[0^{{}^{\circ}}/90^{{}^{\circ}}\right]}_{10}$, $\rho_{s}=1570\;kg/m^{3}$, $R_{1}=20\;mm$, $R_{2}=40\;\mathrm{mm}$, h=2 mm, $L_{2}=120\;\mathrm{mm}$, α=30°, $\eta_{1}^{m}=0.031$, $\eta_{2}^{m}=0.041$, $\eta_{12}^{m}=0.038$, $\eta_{23}^{m}=0.034$, $\eta_{13}^{m}=0.033$ $E_{a}=40\;\mathrm{GPa}$, $E_{b}=10\;\mathrm{GPa}$, $\eta_{a}=0.02498$, $\eta_{b}=0.02555$, $\mu_{a}=0.25$, $\mu_{b}=0.17$, $\rho_{a}=1980\;kg/m^{3}$, $\rho_{b}=1640\;kg/m^{3}$, $h_{c}=0.6\;mm$

## Data Availability

Dataset available on request from the authors.
